# Methods of Using a Manual Defibrillator during Simultaneous Cardiac Arrest in Two Patients—Analysis of the Actions of Emergency Medical Response Teams during the Championships in Emergency Medicine

**DOI:** 10.3390/jcm13185500

**Published:** 2024-09-17

**Authors:** Michał Ćwiertnia, Mieczysław Dutka, Michał Szlagor, Arkadiusz Stasicki, Piotr Białoń, Beata Kudłacik, Maciej B. Hajduga, Monika Mikulska, Mateusz Majewski, Klaudiusz Nadolny, Filip Jaskiewicz, Rafał Bobiński, Marek Kawecki, Tomasz Ilczak

**Affiliations:** 1 Department of Emergency Medicine, Faculty of Health Sciences, University of Bielsko-Biala, Willowa 2, 43-309 Bielsko-Biała, Poland; mszlagor@ubb.edu.pl (M.S.); astasicki@ubb.edu.pl (A.S.); pbialon@ubb.edu.pl (P.B.); bkudlacik@ubb.edu.pl (B.K.); mmikulska@ubb.edu.pl (M.M.); mkawecki@ubb.edu.pl (M.K.); tilczak@ubb.edu.pl (T.I.); 2 European Pre-Hospital Research Network, Nottingham NG11 8NS, UK; 3 Department of Biochemistry and Molecular Biology, Faculty of Health Sciences, University of Bielsko-Biala, Willowa 2, 43-309 Bielsko-Biała, Poland; mdutka@ubb.edu.pl (M.D.); mbhajduga@ubb.edu.pl (M.B.H.); rbobinski@ubb.edu.pl (R.B.); 4 Department of Emergency Medicine, Medical University of Silesia, Ziołowa 45, 40-635 Katowice, Poland; matmaj2@gmail.com; 5 Department of Emergency Medical Service, Faculty of Medicine, Silesian Academy in Katowice, 40-555 Katowice, Poland; prrm.knadolny@interia.pl; 6 Emergency Medicine and Disaster Medicine Department, Medical University of Lodz, 90-419 Lodz, Poland; filip.jaskiewicz@umed.lodz.pl

**Keywords:** advanced life support, cardiac arrest, emergency medicine, defibrillation

## Abstract

**Background/Objectives:** Conducting advanced resuscitation requires medical personnel to carry out appropriately coordinated actions. Certain difficulties arise when it becomes necessary to conduct cardiopulmonary resuscitation (CPR) on two patients at the same time. The aim of this paper was to assess the actions of teams participating in emergency medicine championships in tasks related to simultaneous cardiac arrests in two patients. **Methods:** The study was conducted on the basis of an analysis of assessment cards for tasks carried out during the ‘International Winter Championships in Emergency Medicine’. Three-person medical response teams (MRTs), with the support of two people, had the task of conducting advanced resuscitation on an adult and child simultaneously. The tasks were prepared and developed by European Resuscitation Council (ERC) instructors. **Results:** The study showed that teams used four methods of checking heart rhythm and performing defibrillation during CPR—using paddles only, using paddles and self-adhesive electrodes, using paddles and a three-lead ECG and using two pairs of self-adhesive electrodes. Teams performing cardiopulmonary resuscitation using paddles and a three-lead ECG performed significantly more actions incorrectly than other teams—in part due to the fact that they incorrectly interpreted which patient’s heart rhythm was displayed on the defibrillator screen. The effectiveness of the remaining methods was similar for most of the actions. The CPR method using two pairs of electrodes enabled personal safety to be maintained to the significantly highest percentage during defibrillation. **Conclusions:** The study demonstrated that the need to conduct CPR on two patients at the same time, irrespective of the method used, caused MRT members considerable difficulties in correctly conducting some of the actions. The method of assessing heart rhythm using paddles and a three-lead ECG should not be used. The study showed that the optimal method of CPR in use appears to be the method using two pairs of adhesive electrodes—it provided, among other things, the significantly highest percentage of safely conducted defibrillation.

## 1. Introduction

Treating a patient with sudden cardiac arrest (SCA) requires medical personnel to implement coordinated emergency medical procedures. Of particular significance among these are artificial ventilation, chest compressions and defibrillation [1,2]. Considerable difficulties may arise when medical personnel are providing assistance to two SCA patients simultaneously. In such a situation, depending on the number of people treating the patient, it is necessary to appropriately divide up the emergency medical care actions. In situations where only one defibrillator is available, it becomes particularly problematic to assess the heart rhythm and conduct defibrillation [3].

The need to conduct CPR on two patients at the same time may make it difficult for medical personnel to implement optimal treatment [3]. For this reason, it also seems important for medical personnel to have the opportunity to practice such incidents in the form of simulations as close to real conditions as possible. This is made possible, for example, through participation in emergency medicine championships [4]. For almost 15 years, various types of emergency medicine championships have been held in Poland. One such event is the International Winter Championships in Emergency Medicine, organized by the Bielsko Emergency Services [5].

The subject of the study was taken up because it may have important clinical relevance for patients. It was also important to the authors that due to the low epidemiology of this type of event, there is a lack of scientific reports in this area. For this reason, we undertook an analysis of a simulated situation in which cardiac arrest affected two patients at the same time.

The aim of this paper was to assess the actions of teams participating in the International Winter Championships in Emergency Medicine in tasks related to simultaneous cardiac arrest in two patients. The authors of the research paid particular attention to the methods used by the team members to analyse the patients’ heart rhythm, as well as the way in which they carried out defibrillation.

## 2. Materials and Methods

The research was conducted on the basis of assessment cards for tasks carried out during the International Winter Championships in Emergency Medicine in the years 2013–2023. In these years, tasks related to CPR on child and adult patients were prepared and developed by ERC instructors of European Paediatric Advanced Life Support (EPALS) and Advanced Life Support (ALS). In the period under analysis, there was one task that involved simultaneous cardiac arrest in two patients. This task was conducted during the 11th International Winter Championships in Emergency Medicine, which took place in 2016. In that year, 42 three-person medical response teams made up of paramedics took part in the championship. The research excluded a few guest teams invited from abroad and the organizer’s team, which was not included in the general classification. Directors of emergency services units from around the country could register their current employees for participation in the championship.

In the task that was analysed in detail, the teams received a call to a primary school in which two people had received an electric shock. On arrival, they found two physical education teachers, who were also lifeguards, conducting CPR. One of them was doing chest compressions on an adult and the other on an eight-year-old child. An external automatic defibrillator was not available. The lifeguards had completed certified courses that gave them, among others, the skills and authorization to conduct CPR using a self-inflating bag. With the participation of these two people, the number of personnel was sufficient for advanced resuscitation to be carried out on both patients simultaneously.

According to the championship regulations, each team had a full set of equipment for ALS, including a manual defibrillator. The tasks were carried out in simulated conditions on a MegaCode Kelly manikin for the adult and a MegaCode Kid for the child, both produced by the company Laerdal. The time allocated for carrying out the task was 10 min. The assessment cards used by the judges were developed to assess the compliance of the procedures with ERC guidelines. The judges assessed the following actions carried out by teams on each patient during the tasks: confirmation of SCA before beginning CPR, use of advanced methods for airway management (endotracheal intubation or the use of a supraglottic airway device (SAD)), the heart rhythm when the pulse was checked or not (checking of pulse for ventricular tachycardia (VT) and pulseless electrical activity (PEA), and not checking pulse for ventricular fibrillation (VF) and asystole), the heart rhythm when defibrillation was conducted or not (defibrillating for VF and VT and not defibrillating for PEA and asystole), the method used for conducting defibrillation (shock power levels and personal safety), duration of CPR cycles between subsequent assessments of heart rhythm (for the purposes of the research, the assumed correct cycle duration was from 1:45 to 2:15 min), administering adrenaline (dose, administration route, earlier preparation and the moment when the drug was administered), administering amiodarone (dose, administration route, earlier preparation and the moment when the drug was administered), identifying the heart rhythm for the correct patient (the team’s correct identification of the patient whose heart rhythm is displayed on the defibrillator screen).

During the task, according to the evaluation sheet, the judges evaluated the teams as they performed a certain number of individual actions—counting the total for the child and adult scenario: confirming SCA—2 times, CPR cycles duration—8 times, advanced airway management—2 times, pulse check in case of VT or PEA—6 times, no pulse check in case of VF or asystole—2 times, defibrillation deliver in case of VF or VT—7 times, defibrillation not deliver in case of PEA or asystole—3 times, defibrillation energy—7 times, defibrillation safety—7 times, moment of administering adrenaline—4 times, earlier preparation of adrenaline—4 times, dose of adrenaline—4 times, adrenaline administration route—4 times, moment of administering amiodarone—2 times, earlier preparation of amiodarone—2 times, dose of amiodarone—2 times, amiodarone administration route—2 times, identifying heart rhythm in the correct patient—10 times.

Statistical analysis of the results adopted a level of significance of 0.05. A one-way analysis of variance (ANOVA with Welch’s correction) was used to perform a statistical analysis to determine the procedures for which there were significant differences in the teams’ performance quality, depending on the CPR method used. To check exactly between which CPR methods there were significant differences in the quality of performance of each procedure, a pairwise comparison using Tukey’s post hoc tests was used. Calculations were made using the R statistical environment version 3.6.0, PSPP software version 2.0.0 and MS Office 2019.

## 3. Results

Detailed analysis of the assessment cards and the judges’ remarks on them showed that teams with one defibrillator at their disposal carried out assessments of heart rhythm and defibrillation on patients using four methods:

Method 1 (using only paddles). Teams using this method to assess the first patient’s heart rhythm applied the paddles to his chest and conducted defibrillation when it was required. This action was repeated to assess the heart rhythm in the second patient. This method of assessing heart rhythm and conducting defibrillation was used by 12 teams.

Method 2 (using paddles and self-adhesive electrodes). Teams used this method to assess the first patient’s heart rhythm and conduct defibrillation with self-adhesive electrodes. Assessment of heart rhythm and defibrillation for the second patient were conducted using paddles. Using paddles required the disconnection of the electrodes from the defibrillator and the connection of the paddles. The subsequent heart rhythm assessment of the first patient required disconnection of the paddles and reconnection of the electrodes. This method of assessing heart rhythm and conducting defibrillation was used by nine teams.

Method 3 (using paddles and a three-lead ECG). Teams used this method to assess the first patient’s heart rhythm using a three-lead ECG. If a heart rhythm requiring defibrillation was detected in this patient, the shock was delivered using paddles. Paddles were used to assess the heart rhythm and conduct defibrillation in the second patient. Alternate heart rhythm assessment in the patients required changing the lead each time in the defibrillator settings. This method of assessing heart rhythm and conducting defibrillation was used by eight teams.

Method 4 (using two pairs of self-adhesive electrodes). Teams used this method to assess the first patient’s heart rhythm and conduct defibrillation with self-adhesive electrodes. The second pair of electrodes was used to assess the heart rhythm and conduct defibrillation in the second patient. Alternate heart rhythm assessment in the patients required attaching different electrodes to the defibrillator each time. This method of assessing heart rhythm and conducting defibrillation was used by 13 teams.

All the teams participating in the championships engaged physical education teachers to assist in conducting chest compressions and artificial ventilation.

Detailed research results after statistical preparation are presented in Table 1 and Table 2.

Table 1 presents the significant differences between the methods of carrying out CPR during correctly conducted individual actions. The table also presents information on the number of individual actions performed correctly (C) in relation to the total number of individual actions performed by CPR teams for a particular method (n).

Statistical analysis of the research results showed that significant differences between the methods of conducting CPR were noted in the case of 11 actions. Table 2, developed using a pairwise comparison using Tukey’s post hoc test, shows the exact CPR methods between which there were significant differences in carrying out individual actions correctly.

The research showed that the significantly highest percentage of correct duration of CPR cycles was noted during CPR conducted using method 1 and the lowest during CPR conducted using methods 2 and 3. There was also a significant difference between method 1, which had the highest percentage, and method 2, which had the lowest percentage of the use of advanced methods of airway management.

The results of the study showed that teams performing CPR by methods 1, 2 and 4 obtained a significantly higher percentage of seven correctly performed actions than teams performing CPR by method 3. These actions were: pulse check in case of VT or PEA, no pulse check in case of VF or asystole, defibrillation delivered in case of VF or VT, defibrillation not delivered in case of PEA or asystole, moment of administering adrenaline, moment of administering amiodarone and identifying heart rhythm in the correct patient. In terms of the correct conduct of these actions, no significant differences were noted between the results obtained by the teams conducting CPR with methods 1, 2 and 4.

An analysis of defibrillation delivery showed that conducting defibrillation safely was noted at a significantly higher percentage for CPR conducted using method 4 than for CPR conducted using the other methods. No significant differences were noted in the percentages of conducting defibrillation safely between methods 1, 2 and 3.

The research showed that the administration of amiodarone at the correct dose was noted at a significantly higher percentage for CPR conducted using methods 1 and 4 than for CPR conducted using method 3. No significant differences were noted in the percentages of administering amiodarone at the correct dose between methods 2 and 3, or between methods 1, 2 and 4.

## 4. Discussion

Members of MRTs should begin CPR once SCA has been confirmed in a patient [6,7,8]. This action should also be taken when an MRT arrives and takes over resuscitation from witnesses present at the scene of the incident. This also has the aim of avoiding situations in which CPR is conducted on a patient without cardiac arrest [9]. This research has shown that, depending on the method of conducting CPR, MRT members confirmed SCA in 66.67% to 69.23% of cases. The relatively high percentage of errors may result from the incorrect assumption by MRT members that if CPR is already being conducted by incident witnesses, then there is no need to confirm SCA. No significant differences were shown in this respect between the methods analysed.

According to ERC guidelines, medical personnel should conduct ALS in two-minute cycles between subsequent heart rhythm assessments [1,9]. During the development of the assessment cards, it was assumed that the correct CPR cycle duration would be 1:45–2:15 min. The research has shown that the MRTs obtained a relatively low percentage of correct CPR cycle duration for each of the methods (from 34.38% to 43.75%). In other research into the analysis of the actions of MRTs during the emergency care championships, a higher percentage was noted in this respect (from 48.40% to 61.23%) [5]. Most certainly, the lower results in this research were influenced by the fact that MRT members had to monitor the cycle duration in two patients simultaneously. The research has also shown that the significantly highest percentage of correct CPR cycle duration was noted in teams conducting CPR using method 1. The significantly lower percentage in the duration of cycles using the remaining methods could result from the need to carry out additional actions, such as changing leads and connecting electrodes and paddles.

The correct method of airway management enables effective ventilation to be conducted in patients with SCA [10,11,12]. Advanced airway management using endotracheal intubation or SAD brings additional benefits. These include the possibility of conducting asynchronous CPR [9,13], the possibility of using capnography [14,15] and the lower risk of stomach distension and gastric aspiration into the airway [16]. This research has shown that teams conducting CPR using method 2 used a significantly lower percentage of advanced airway management than those using method 1.

During CPR, with subsequent assessments of heart rhythm, pulse checks should either be carried out or omitted. The pulse should be checked when a heart rhythm appears with visible QRS complexes. This allows for the return of spontaneous circulation (ROSC) to be identified during CPR. The pulse should not be checked in the case of VF or asystole. Conducting pulse assessments at these heart rhythms causes unnecessary pauses in chest compressions [9,13]. As shown in numerous research studies [17,18,19], pauses in chest compressions lower the chances of ROSC and the survival of patients with SCA. This research has shown that teams conducting CPR using method 3 obtained a significantly lower percentage of correct actions in terms of pulse checking or omitting at various heart rhythms. No significant differences were noted in this respect between methods 1, 2 and 4.

Depending on the identified heart rhythm, MRT members should either conduct or omit defibrillation. Defibrillation should be delivered immediately to a patient with VF or pulseless VT [9,13]. Numerous studies have shown that such action significantly increases the chances of survival for patients with SCA [20,21,22]. Defibrillation should not be conducted for PEA and asystole. Delivering shocks at these heart rhythms can cause myocardial damage [23] and generate additional pauses in chest compressions [9]. This research has shown that teams conducting CPR using method 3 acted incorrectly with respect to conducting or omitting defibrillation significantly more often than teams conducting CPR using the other three methods. No significant differences were noted in this respect between methods 1, 2 and 4.

According to the 2015 ERC guidelines in force in 2016, all defibrillations on children should be carried out with an energy of 4 J/kg of body mass [24], while for adults they should be conducted with energies according to the manufacturer’s recommendations [9]. Research has shown that the use of the correct energy for shocks increases the chances of restoring sinus rhythm [25,26]. Our research has shown that the percentage of defibrillations carried out with the correct energy setting for various methods ranged between 85.71% and 87.91%. Errors committed by teams in this respect most certainly could have been related to the need to change the power setting between that needed for an adult and that needed for a child. No additional significant differences were found between the different methods in the percentage of correct completion of this action.

The most important aspect of actions related to ALS conducted by medical personnel is maintaining safety [27,28,29]. A particularly important moment in this respect occurs during defibrillation. Cases have been recorded of medical personnel [30] and patients [31] suffering serious injuries due to a lack of appropriate safety measures during the procedure. Soar et al. [9] noted that the use of electrodes instead of paddles can be useful in maintaining a suitable degree of safety during defibrillation. This research has shown that teams conducting CPR with methods that use paddles (methods 1, 2 and 3) conducted defibrillation safely at a significantly lower percentage than teams using only electrodes (method 4). Analysis of the judges’ remarks on the assessment cards showed that the most common error in carrying out this procedure involved one of the five people conducting CPR touching the patient while shocks were being delivered. In the remaining cases, it was noted that the source of oxygen was not removed to a safe distance.

One of the elements of conducting ALS is the use of pharmacotherapy. Research has shown that the correct administration of adrenaline increases the chances of survival for patients with SCA [32,33]. The adrenaline dosage for adults is 1 mg, and for children it is 0.01 mg per kg of body mass. The drug should be administered intravenously or intraosseously as a bolus. Earlier preparation of the adrenaline facilitates its administration [1]. Our research has shown that adrenaline was always administered by the teams via the correct route, and in the vast majority of cases, in the correct dose. Incorrect doses were, in every case, given during the administration of the drug to a child. For all methods of conducting CPR, there was a relatively low percentage of adrenaline administered at the correct time (from 53.13% to 59.61%), as well as of its earlier preparation (from 46.86% to 48.07%). The frequent errors in this respect were most certainly influenced by the need to coordinate the administration of the drug to two patients simultaneously. With regard to the administration of adrenaline, it was shown that teams conducting CPR using methods 1, 2 and 4 administered adrenaline at the correct time significantly more often than teams conducting CPR using method 3.

According to ERC guidelines, patients with refractory shockable rhythms should immediately be given amiodarone. For adults, this drug should be given after the third defibrillation at a dose of 300 mg and after the fifth defibrillation at a dose of 150 mg. In children, amiodarone is also given after the third and fifth defibrillations at a dose of 5 mg/kg of body mass. In a patient with SCA, amiodarone should be given intravenously or intraosseously as a bolus. Earlier preparation of this drug facilitates its administration at the correct time [1]. This research has shown that, similar to the administration of adrenaline, amiodarone was always given via the correct route, and the few cases of the incorrect dose being given always involved children. The drug was prepared earlier in individual methods in 43.75% to 46.15% of cases and was administered at the correct time in 62.50% to 73.07% of cases. The administration of amiodarone at the incorrect time was most certainly influenced by the need for team members to remember the number of defibrillations already conducted on each patient. In terms of the administration of amiodarone, it was shown that teams conducting CPR using methods 1, 2 and 4 gave amiodarone at the correct time significantly more frequently than teams conducting CPR using method 3. It was also shown that teams conducting CPR using methods 1 and 4 gave the correct dose of amiodarone significantly more frequently than teams conducting CPR using method 3.

It is extremely important that MRT members are able to correctly assess the heart rhythm of a patient with SCA, as this has an impact on further decisions related to, among other things, defibrillation, pulse check and pharmacotherapy [9,13]. Detailed analysis conducted during this research of the assessment cards and the judges’ remarks included on them showed that, in some cases, teams conducting CPR using method 3 incorrectly identified the patient whose heart rhythm was displayed on the defibrillator screen. As demonstrated, this had a direct impact on there being more frequent errors than in the other methods in defibrillation (conducting defibrillation on the wrong patient or not conducting defibrillation on a patient with a heart rhythm requiring defibrillation), pulse check (pulse check on a patient with VF and asystole, or omission of pulse check on a patient with PEA and VT), and pharmacotherapy (incorrectly determined time for administering adrenaline or amiodarone). This type of error occurred when one of the patients was being monitored with the use of a three-lead ECG with their heart rhythm displayed on the defibrillator screen. If at this moment a member of the MRT placed the paddles on the chest of the second patient without changing the leads in the defibrillator settings, they assumed that the heart rhythm visible on the monitor belonged to the patient on whose chest the paddles had been placed.

Performing CPR on two patients at the same time can be a big challenge for medical personnel [3]. Our studies have shown that the method of managing the manual defibrillator in this situation can significantly affect the quality of the procedures performed during CPR. Although such situations are very rare and medical personnel may not have experience with such cases, effective management by the resuscitation team can lead to the survival of both patients [34]. Due to the clinical significance and lack of scientific reports in this area, it is important to design future studies to create effective procedures for resuscitating two patients at the same time.

## 5. Conclusions

The research, conducted in simulated conditions, showed that the need to conduct CPR on two patients at the same time, irrespective of the method used, caused the members of MRTs considerable difficulties in correctly carrying out some of the actions. Based on the results, the authors of this study suggest that the method employed by some of the teams to assess heart rhythm using paddles and three-lead ECG readings (method 3) should not be used. As demonstrated, this method brings with it the risk of identifying the heart rhythm of the wrong patient. The research results indicate that the best of the methods used would appear to be using two pairs of self-adhesive electrodes (method 4). In the majority of cases, this method provided a comparable quality of actions as using only paddles (method 1) and using paddles and self-adhesive electrodes (method 2). A key factor, however, would seem to be the fact that this method ensured the significantly highest percentage of defibrillations conducted in a safe manner. We suggest that training of medical personnel should include in the program simulated scenarios of performing CPR on two patients at the same time. It may also be important that defibrillators used by medical personnel have more than one pair of adhesive electrodes in their equipment.

## Figures and Tables

**Table 1:** Presentation of significant differences between the methods of carrying out CPR during correctly conducted individual actions.

Correctly Conducted Individual Actions		CPR Methods				F	*p*
		1 (12 Teams)	2 (9 Teams)	3 (8 Teams)	4 (13 Teams)		
Confirming SCA	C/n %	16/24 66.67%	12/18 66.67%	11/16 68.75%	18/26 69.23%	1.497	0.228
CPR cycle duration	C/n %	42/96 43.75%	25/72 34.72%	22/64 34.38%	41/104 39.42%	16.040	<0.001
Advanced airway management	C/n %	20/24 83.33%	14/18 77.78%	13/16 81.25%	21/26 80.77%	4.280	0.009
Pulse check in case of VT or PEA	C/n %	61/72 84.72%	45/54 83.33%	34/48 70.83%	66/78 84.62%	36.591	<0.001
No pulse check in case of VF or asystole	C/n %	22/24 91.67%	16/18 88.89%	13/16 81.25%	23/26 88.46%	17.096	<0.001
Defibrillation delivered in case of VF or VT	C/n %	76/84 90.48%	57/63 90.48%	41/56 73.21%	82/91 90.11%	60.765	<0.001
Defibrillation not delivered in case of PEA or asystole	C/n %	34/36 94.44%	25/27 92.59%	17/24 70.83%	37/39 94.87%	110.091	<0.001
Defibrillation energy	C/n %	72/84 85.71%	55/63 87.30%	49/56 87.50%	80/91 87.91%	0.759	0.523
Defibrillation safety	C/n %	73/84 86.90%	54/63 85.71%	48/56 85.71%	86/91 94.51%	14.674	<0.001
Moment of administering adrenaline	C/n %	28/48 58.33%	21/36 58.33%	17/32 53.13%	31/52 59.61%	6.761	<0.001
Earlier preparation of adrenaline	C/n %	23/48 47.91%	17/36 47.22%	15/32 46.86%	25/52 48.07%	0.267	0.849
Dose of adrenaline	C/n %	44/48 91.67%	32/36 88.89%	29/32 90.63%	47/52 90.38%	1.074	0.369
Adrenaline administration route	C/n %	48/48 100%	36/36 100%	32/32 100%	52/52 100%	0.000	1.000
Moment of administering amiodarone	C/n %	17/24 70.83%	13/18 72.22%	10/16 62.50%	19/26 73.07%	19.278	<0.001
Earlier preparation of amiodarone	C/n %	11/24 45.83%	8/18 44.44%	7/16 43.75%	12/26 46.15%	1.053	0.378
Dose of amiodarone	C/n %	22/24 91.67%	16/18 88.89%	14/16 87.50%	24/26 92.31%	4.239	0.010
Amiodarone administration route	C/n %	24/24 100%	18/18 100%	16/16 100%	26/26 100%	0.000	1.000
Identifying heart rhythm in the correct patient	C/n %	120/120 100%	90/90 100%	69/80 86.25%	130/130 100%	38.600	<0.001

Method 1—using only paddles, method 2—using paddles and self-adhesive electrodes, method 3—using paddles and three-lead ECG, method 4—using two pairs of self-adhesive electrodes, C—number of procedures performed correctly, n—total number of procedures performed, F—test statistic (a one-way analysis of variance (ANOVA with Welch’s correction)), *p*—statistical significance (*p* < 0.05).

**Table 2:** Presentation of methods of conducting CPR between which there were significant differences in carrying out individual actions correctly.

Correctly Conducted Individual Actions		Method 2	Method 3	Method 4
CPR cycle duration	Method 1	*p* < 0.001	*p* < 0.001	*p* = 0.031
	Method 2	-	*p* = 0.996	*p* = 0.016
	Method 3		-	*p* = 0.008
Advanced airway management	Method 1	*p* < 0.003	*p* = 0.535	*p* = 0.352
	Method 2	-	*p* = 0.119	*p* = 0.220
	Method 3		-	*p* = 0.989
Pulse check in case of VT or PEA	Method 1	*p* = 0.804	*p* < 0.001	*p* = 1.000
	Method 2	-	*p* < 0.001	*p* = 0.837
	Method 3		-	*p* < 0.001
No pulse check in case of VF or asystole	Method 1	*p* = 0.280	*p* < 0.001	*p* = 0.597
	Method 2	-	*p* < 0.001	*p* = 0.946
	Method 3		-	*p* < 0.001
Defibrillation deliver in case of VF or VT	Method 1	*p* = 1.000	*p* < 0.001	*p* = 0.995
	Method 2	-	*p* < 0.001	*p* = 0.995
	Method 3		-	*p* < 0.001
Defibrillation not deliver in case of PEA or asystole	Method 1	*p* = 0.629	*p* < 0.001	*p* = 0.992
	Method 2	-	*p* < 0.001	*p* = 0.455
	Method 3		-	*p* < 0.001
Defibrillation safety	Method 1	*p* = 0.867	*p* = 0.867	*p* < 0.001
	Method 2	-	*p* = 1.000	*p* < 0.001
	Method 3		-	*p* < 0.001
Moment of administering adrenaline	Method 1	*p* = 1.000	*p* < 0.001	*p* = 0.992
	Method 2	-	*p* < 0.001	*p* = 0.455
	Method 3		-	*p* < 0.001
Moment of administering amiodarone	Method 1	*p* = 0.804	*p* < 0.001	*p* = 0.471
	Method 2	-	*p* < 0.001	*p* = 0.946
	Method 3		-	*p* < 0.001
Dose of amiodarone	Method 1	*p* = 0.280	*p* < 0.041	*p* = 0.976
	Method 2	-	*p* = 0.804	*p* = 0.127
	Method 3		-	*p* < 0.013
Identifying heart rhythm in the correct patient	Method 1	*p* = 1.000	*p* < 0.001	*p* = 1.000
	Method 2	-	*p* < 0.001	*p* = 1.000
	Method 3		-	*p* < 0.001

Method 1—using only paddles, method 2—using paddles and self-adhesive electrodes, method 3—using paddles and three-lead ECG, method 4—using two pairs of self-adhesive electrodes, *p*—statistical significance (*p* < 0.05).

## Data Availability

The datasets used and/or analysed during the current study are available from the corresponding author upon reasonable request.
